# Supplementary material for: Complexome profiling of the Chlamydomonas psb28 mutant reveals TEF5 as an early PSII assembly factor
Source: Plant Cell. 2025 Mar 17;37(6):koaf055. doi: 10.1093/plcell/koaf055 (PMC12164590; doi:10.1093/plcell/koaf055)
Supplement: koaf055_Supplementary_Data [file koaf055_supplementary_data.zip › psb28_interactive.zip!/plots/intensity/qualityPlots/peptides/LHCA5_Cre10.g425900.t1.2.html]

### Cre10.g425900.t1.2

Trivial name: LHCA5  
SeqCoverage [median]: 54.90  
Mass: 28.23 kDa  
  
 

| Sequence | MissedCleavage | Mass [Da] | wtIntensity | muIntensity | Score |
| --- | --- | --- | --- | --- | --- |
| EIKNGRLAMIAYMAFILQAQATGK | 2 | 2637.41 | 3323.78 | 0.00 | 72.14 |
| GDLAGDYGWDPLGLGADPTALK | 0 | 2201.06 | 139155.68 | 0.00 | 219.41 |
| GNKVPNPEMGYPGGIFDPFGFSK | 1 | 2454.17 | 333808.24 | 298266.05 | 133.51 |
| GNLKELQTK | 1 | 1029.58 | 534428.29 | 468803.32 | 332.05 |
| GPLAALSAHLSNPFGNNILK | 0 | 2033.11 | 264608.49 | 348602.72 | 223.11 |
| KLWAPGVVAPEYLK | 1 | 1569.89 | 183258.79 | 220409.68 | 109.84 |
| LAMIAYMAFILQAQATGK | 0 | 1940.03 | 289761.00 | 136299.95 | 195.60 |
| LAMIAYMAFILQAQATGKGPLAALSAHLSNPFGNNILK | 1 | 3955.12 | 2431.23 | 0.00 | 47.04 |
| LWAPGVVAPEYLK | 0 | 1441.80 | 192095.97 | 37537.29 | 141.29 |
| NFGSVNEDPIFK | 0 | 1365.66 | 1006298.66 | 1236614.22 | 276.00 |
| NFGSVNEDPIFKGNK | 1 | 1664.82 | 56522.11 | 63334.79 | 195.10 |
| NFGSVNEDPIFKGNKVPNPEMGYPGGIFDPFGFSK | 2 | 3801.81 | 29083.92 | 0.00 | 69.49 |
| QSELQHAR | 0 | 967.48 | 158465.24 | 80950.80 | 243.54 |
| RWQDYKNFGSVNEDPIFK | 2 | 2242.08 | 1861.60 | 1035.51 | 31.82 |
| VPNPEMGYPGGIFDPFGFSK | 0 | 2155.01 | 208949.40 | 152867.75 | 168.05 |
| WQDYKNFGSVNEDPIFK | 1 | 2085.98 | 62818.38 | 32896.87 | 170.23 |
| WQDYKNFGSVNEDPIFKGNK | 2 | 2385.14 | 2500.33 | 0.00 | 50.43 |
|  |  |  | 3469371.1 | 3077619.0 |  |
